# Defying distance: exercise providers’ perspectives on remote physical activity supports for older adults: a mixed-methods study

**DOI:** 10.3389/fpubh.2025.1643477

**Published:** 2025-10-06

**Authors:** Samira Mehrabi, Sara Drisdelle, Hanna R. Dutt, Laura E. Middleton

**Affiliations:** ^1^Department of Kinesiology and Health Sciences, University of Waterloo, Waterloo, ON, Canada; ^2^Schlegel-UW Research Institute for Aging, Waterloo, ON, Canada

**Keywords:** physical activity, older adults, remote supports, virtual exercise, technology, home-based exercise, health promotion, mixed-methods

## Abstract

**Introduction:**

Many older adults encounter barriers to participating in physical activity programs, often due to cost, accessibility, and transportation challenges. Implementing feasible and effective remote support strategies may enhance their physical activity participation. This mixed-methods study examines exercise providers’ use of remote supports for physical activity among older adults, their perceived effectiveness, and barriers and facilitators to adoption during the COVID-19 pandemic and beyond.

**Methods:**

Exercise providers (≥18 years) completed a web-based survey (June–September 2020), and optional semi-structured interviews (September–December 2020), guided by the COM-B model. Participant characteristics, uptake and perceived effectiveness of remote supports, and presence and severity of barriers were explored and analyzed with inductive thematic analysis.

**Results:**

Fifty-one exercise providers (age 36.3 ± 12.3 years, 38 female) completed the survey; 86% provided remote support for physical activity, including provision of copy materials (63%) and delivery of real-time virtual programs (59%), with the latter rated the most effective (88%). Key barriers included older adults’ limited technical skills (78%) and access to technology (82%). Interviews (*n* = 12, age 40.5 ± 15 years, 11 female), yielded five themes: (1) Capacity, Collaboration, and Adaptability Supported Successful Transition to Remote Supports; (2) Tailoring Remote Supports to Needs and Abilities Promoted Safety; (3) Real-time Virtual Programs Fostered Social Support and Engagement; (4) Accessible Technology and Ongoing Support Facilitated Virtual Delivery; and (5) A Hybrid Approach Balances Convenience and Social Benefits.

**Conclusion:**

During the transition to virtual exercise programming during the COVID-19 pandemic, exercise providers widely used remote supports, favoring real-time virtual programs for socialization and supervision. While there were challenges including safety concerns, technological barriers, and engagement, these challenges were met with innovative solutions. A hybrid approach may be the most sustainable model, balancing the accessibility of virtual programs with the social and motivational benefits of in-person exercise.

## 1 Introduction

Engagement in physical activity is essential for maintaining physical health and functional abilities, reducing the risk of chronic health conditions, and managing pre-existing conditions (1–5). Regular participation in physical activity can also improve psychological well-being (6–8), cognitive function (9–11), and quality of life (3, 6) in older adults. However, there are many barriers to participating in exercise programs in-person, including limited access to facilities, high program costs, and time constraints due to work responsibilities, caregiving, or competing demands (12–15). Poor weather, safety concerns and limited access to transportation further restrict physical activity opportunities for older adults, particularly in rural areas (12, 14–19). Using public transport can be particularly challenging for frail older adults or those with cognitive impairment (17).

Remote supports for physical activity offer a promising solution to overcoming barriers to physical activity for older adults (18–20). Technologies such as web-based communication and video conferencing enable flexible program delivery, supporting home-based participation (21). This flexibility enhances accessibility, allowing older adults to engage in physical activity from the comfort of their homes (20, 22, 23).

The COVID-19 pandemic heightened demand for remote physical activity supports as public health restrictions and social distancing measures closed exercise facilities and reduced physical activity levels especially among older adults (24, 25). This urgency drove the rapid development and implementation of remote strategies to counter physical inactivity risks (26–28). Accelerated technology adoption positioned technology-based interventions for physical activity as viable and sustainable options (28–33). Exercise providers explored various technologies and delivery methods, assessing their effectiveness and practicality for long-term use (32–34).

While the existing literature largely focuses on older adults’ experiences with remote interventions (22, 35, 36), exercise providers’ perspectives on implementing these strategies remain relatively underexplored (35, 36). Understanding the facilitators and barriers that exercise providers encountered during the delivery of remote supports for physical activity is crucial for scalability and sustainability of these supports, particularly as older adults increasingly embrace technology-based solutions for health and well-being (37, 38). To address this gap, this study aimed to: (i) describe remote supports for physical activity provided to older adults early in the COVID-19 pandemic; (ii) explore exercise providers’ perceptions of their effectiveness; (iii) identify facilitators and barriers to delivery of remote supports during the pandemic; (iv) assess implications for future programming and sustainability.

## 2 Materials and methods

### 2.1 Study design

This study used an explanatory, sequential, mixed-methods design, with both quantitative and qualitative data collection methods. Qualitative and quantitative methods were given equal weight and their findings were integrated for interpretation using a contiguous approach, meaning that the results are presented in a single report with the qualitative and quantitative findings reported separately (39). The data collection process started with the administration of a web-based survey to collect quantitative data, followed by semi-structured one-on-one interviews to collect qualitative data. The survey was open from June 12, 2020, to September 23, 2020, and interviews were conducted between October 2020 to January 2021. This study was approved through a University of Waterloo human ethics committee (#42191). All participants provided electronic consent (survey) or verbal consent (interviews).

### 2.2 Study sample and recruitment

Exercise providers were eligible for the study if they: (i) were 18 years and older; (ii) had experience supporting older adults’ physical activity pre-pandemic; (iii) were English- speaking; and (iv) had access to the internet and an appropriate device (e.g., computer, tablet, smartphone).

Participants were recruited via convenience and snowball sampling through social media (e.g., Facebook, Twitter, and LinkedIn), professional networks, community and fitness centers’ mailing lists, outreach to relevant organizations (e.g., fitness centers, physical therapy), and word-of-mouth.

### 2.3 Web-based survey

Participants completed a survey via the Qualtrics [Provo, UT] (40), assessing demographics (age, gender, ethnicity, country, marital status) and self-rated physical and mental health (5-point Likert scale: poor to excellent). Other questions captured certifications and practice: (i) occupation (i.e., physiotherapist, kinesiologist, exercise physiologist, personal trainer, group fitness instructor, manager/owner of a fitness/rehabilitation facility); (ii) qualifications (e.g., registered kinesiologist, certified exercise physiologist); (iii) years of experience; and (iv) delivery location (urban vs. rural). Questions also addressed clientele (e.g., older adults, chronic conditions) and pre-COVID delivery methods (in-person vs. virtual).

The survey also examined the adoption of remote strategies to support physical activity during pandemic closures with perceived effectiveness rated on 4-point Likert scale (not effective to very effective). Perceived barriers to remote supports activity were rated on a 5-point Likert scale (not limiting to extremely limiting). Additionally, the survey explored technology access and knowledge, virtual platform experience, and transition time to remote programming.

### 2.4 Semi-structured interviews

Survey respondents who indicated interest in completing interviews received email invitations. Interviews were conducted via phone or on a video conferencing platform, as per the participant’s preference. The interviews were guided by COM-B model (i.e., capability, opportunity, and motivation) (41) and explored facilitators and barriers associated to remote supports for older adults’ physical activity within these domains with a specific focus on technology-reliant strategies.

### 2.5 Data analysis

#### 2.5.1 Quantitative

Due to the study’s exploratory design, no formal sample size calculation was performed. Descriptive statistics were calculated for participant characteristics and presented as means ± standard deviation (SD), median (range), or number and percentages (*n* (%)), as appropriate.

#### 2.5.2 Qualitative

We adopted a post-positivist perspective, which holds the existence of a single, objective reality that can be understood to a certain extent through empirical observations and rigorous scientific methods (42, 43). While we made a conscious effort to maintain objectivity throughout the data collection and analysis, we also acknowledged that our understanding and interpretation were shaped by personal biases and assumptions, as well as our academic and non-academic experiences (42, 43). In this case, all authors were women in the Department of Kinesiology and Health Sciences at various levels (two graduate students, one undergraduate student, and one faculty member) at the time of the data collection and analysis.

Interviews (mean 47.3 ± 12 min) were digitally recorded and transcribed verbatim. The transcripts were checked, cleaned, de-identified, and analyzed in NVivo (version 13; QSR International) (44). Inductive thematic analysis, guided by Braun and Clarke’s 6-phase framework (45), was conducted by the primary researcher (SM) and two student research assistants to identify key topics and patterns across the interviews. Initially, the team immersed themselves in the data through repeated readings of the transcripts, followed by independent line-by-line coding. A preliminary codebook was iteratively developed and refined by the coding team to ensure accuracy and comprehensiveness. Codes were then collated into initial themes and sub-themes, which were further refined in consultation with the senior researcher (LEM) for coherence and distinction (45). Themes without enough supporting data or themes that were too diverse were revised, merged, or discarded. Finally, the refined themes and sub-themes were named and detailed, with illustrative quotes drawn from the data to encapsulate their essence (45).

To ensure the rigor and trustworthiness of the study, the team met frequently throughout the analysis process to debrief, review, refine the codebooks and emerging themes/sub-themes, and resolve any major discrepancies or challenges (42, 46–49). An audit trail was established by preserving the audio recordings of the interviews, field notes and debriefing notes, and reflexive memoing throughout the study. Additionally, all stages of the study were clearly documented and described in detail, including in-depth descriptions of the research methods, the setting, and the data collected, as well as a comprehensive and thorough account of the research findings.

## 3 Results

### 3.1 Participants

Fifty-one exercise providers (age 36.3 ± 12.3 years) completed the survey; 74% were female with 82 and 59% reporting very good-to-excellent physical health and mental health, respectively. Participants had an average of 9.5 years of experience delivering exercise, with 80% holding at least one exercise related certification or professional designation and 86% providing remote physical activity support to older adults during the pandemic.

Of thirty survey respondents who expressed interest in a follow-up interview, twelve ultimately participated (5 by phone, 7 via Zoom). The remaining individuals did not respond to follow-up communication. Interviewees had a mean age of 40.5 ± 15.0 years, with 92% identifying as female. They averaged 8 years of work experience, and 83% held an exercise provider certification or professional designation. All resided in Canada and reported very good or excellent physical and mental health. Participant characteristics are presented in Table 1.

**Table 1:** Participant characteristics (mean (SD) or *n* (%)).

Characteristic	Survey (*n* = 51)	Interview (*n* = 12)
Age, years	36.3 (12.3)	40.5 (15)
Gender		
Female	38 (74.5%)	11 (92%)
Male	12 (23.5%)	1 (8%)
Prefer not to disclose	1 (2%)	0 (0.0%)
Country of residence, Canada	39 (76.5%)	12 (100%)
Self-rated physical health, ≥ very good	42 (82.4%)	12 (100%)
Self-rated mental health, ≥ very good	30 (58.8%)	12 (100%)
Work experience, years	9.5 (8.6)	8 (7.3)
Role		
Kinesiologist	14 (27.4%)	2 (16.6%)
Group fitness instructor/personal trainer	13 (25.4%)	6 (50%)
Physiotherapist	10 (19.6%)	2 (16.6%)
Exercise physiologist	8 (15.6%)	2 (16.6%)
Occupational therapist	2 (4%)	0 (0.0%)
Other	4 (8%)	0 (0.0%)
Exercise delivery during COVID-19, yes	44 (86.3%)	12 (100%)
Location of exercise delivery		
Urban	44 (86.3%)	10 (83.3%)
Rural	4 (8%)	2 (16.6%)
Both	2 (4%)	0 (0.0%)
Prefer not to disclose	1 (2%)	0 (0.0%)

### 3.2 Quantitative results

#### 3.2.1 Remote physical activity supports during the pandemic

Before the pandemic, most providers offered no remote exercise programming or services (median 0%). In contrast, during the survey period, 96% of programming and supports shifted to remote delivery, with a median transition time of 2 weeks (range: 0–12 weeks). The most common remote supports included hard copy materials (63%) and real-time virtual programs (59%). The use of other supports is described in Table 2.

**Table 2:** Provided remote physical activity supports and their perceived effectiveness (% (*n*)).

Physical activity supports		Perceived effectiveness			
Provided by exercise providers	Very effective	Moderately effective	Slightly effective	Not effective
Hard-copy exercise programs/instruction	63.6% (28)	0% (0)	42.9% (12)	53.6% (15)	3.6% (1)
Real-time virtual exercise programs	59.0% (26)	42.3% (11)	46.2% (12)	11.5% (3)	0% (0)
E-mailed exercise programs/instruction	56.8% (25)	8.0% (2)	48.0% (12)	44.0% (11)	0% (0)
Phone/web-chat check-ins	52.2% (23)	13.0% (3)	56.5% (13)	26.1% (6)	4.3% (1)
Information for at-home exercise	45.4% (20)	0% (0)	30.0% (6)	60.0% (12)	5.0% (1)
Phone/web-chat instruction	34.9% (15)	20.0% (3)	46.7% (7)	20.0% (3)	6.7% (1)
Pre-recorded exercise videos	29.5% (13)	7.7% (1)	46.2% (6)	46.2% (6)	0% (0)

All exercise providers reported access to a computer (desktop or laptop) or mobile device (e.g., tablet, smartphone). Zoom was the most used platform (41%) for virtual exercise programming, with sessions averaging 45 min (range 10–60 min).

#### 3.2.2 Perceived effectiveness of remote physical activity supports during the pandemic

Table 2 summarizes the perceived effectiveness of remote supports. Hard-copy materials were perceived as least effective, rated “not effective” or “slightly effective” by 57% of providers. Real-time virtual programming was considered most effective, with 88% rating it “moderately effective” or “very effective.” Phone or web-chat check-ins and instructions were viewed as “moderately effective” or “very effective” by two-thirds (68%) of providers.

#### 3.2.3 Perceived barriers to remote physical activity support during the pandemic

Table 3 summarizes exercise providers perceived barriers to remote support of physical activity during the early stage of the pandemic. Most providers rated older adults’ limited technology access (82%) and lack of technical skills (78%) as “moderately limiting” or “extremely limiting.” Nearly half (47%) also viewed older adults decreased mental health and lack of at-home exercise equipment as “moderately limiting” or “extremely limiting.”

**Table 3:** Perceived barriers to remote physical activity supports (% (*n*)).

Limiting factor	Extremely limiting	Very limiting	Moderately limiting	Slightly limiting	Not limiting
Older adults limited technological skills	39.2% (20)	25.5% (13)	13.8% (7)	11.8% (6)	9.8% (5)
Older adults limited access to technology	37.3% (19)	27.5% (14)	17.6% (9)	9.8% (5)	7.8% (4)
Concerns about the safety of unsupervised exercise	13.8% (7)	25.5% (13)	37.3% (19)	17.6% (9)	5.8% (3)
Older adults decreased mental wellness	9.8% (5)	37.3% (19)	31.4% (16)	11.8% (6)	9.8% (5)
Older adults limited access to equipment or inadequate exercise space	9.8% (5)	37.3% (19)	31.4% (16)	11.8% (6)	9.8% (5)
Lack of technological skills	7.8% (4)	9.8% (5)	19.6% (10)	21.6% (11)	41.1% (21)
Competing demands	5.9% (3)	19.6% (10)	15.7% (8)	15.7% (8)	43.1% (22)
Decreased mental wellness	3.9% (2)	15.7% (8)	13.8% (7)	23.5% (12)	43.1% (22)
Lack of technological resources	3.9% (2)	13.7% (7)	11.7% (6)	21.5% (11)	49.0% (25)
Limited contact information for clients	2.0% (1)	17.6% (9)	31.4% (16)	17.6% (9)	31.3% (16)
Lack of confidence providing exercise remotely/virtually	3.9% (2)	7.8% (4)	25.5% (13)	11.8% (6)	50.9% (26)
Concerns about privacy or security of online platforms	3.9% (2)	2.0% (1)	31.4% (16)	23.5% (12)	39.2% (20)

### 3.3 Qualitative results

Five themes were identified from interviews of exercise providers: (1) Capacity, Collaboration, and Adaptability Supported Successful Transition to Remote Supports; (2) Tailoring Remote Supports to Needs and Abilities Promoted Safety; (3) Real-time Virtual Programs Fostered Social Support and Engagement; (4) Accessible Technology and Ongoing Support Facilitated Virtual Delivery; and (5) A Hybrid Approach Balances Convenience and Social Benefits. The Overview Table presents a summary of practical recommendations that emerged from interviews, highlighting strategies to enhance virtual program delivery. These recommendations were derived directly from participants’ descriptions of effective practices based on their experience. During thematic analysis, examples and actionable tips were extracted, clustered by topic (e.g., onboarding, safety, program design and delivery), and synthesized into concise, actionable recommendations while preserving the original intent of the participants.

#### 3.3.1 Theme 1: capacity, collaboration, and adaptability supported successful transition to remote supports

The rapid shift to remote supports required significant adaptability by exercise providers. Many providers quickly adopted virtual delivery during the pandemic, shifting from basic resources (e.g., hard-copy materials) to comprehensive real-time virtual programs or pre-recorded videos as organizational support and resources expanded. Some providers found the transition relatively seamless due to their familiarity with home-based exercise programming: *“As a physio, I’m used to giving patients detailed home exercise programs, so to me that [virtual programming] did not seem like too big of a transition.” (Physiotherapist)* Another provider highlighted how familiarity with virtual platforms was an asset for delivering virtual exercise during the pandemic:

*When the pandemic hit and we were going to be at home, right away I realized that our seniors would quickly lose their mobility, so I touched base with my supervisor and said to him, “I’d like to use this Facebook page that I’d been working on to try the fitness classes there.” (Group fitness instructor).*

For others, success depended on organizational support, technical resources, and collaboration. Exercise providers in well-resourced settings benefited from extra staffing, technical assistance, and access to established virtual platforms using technical support teams for platform setup and software management. In contrast, those in resource-limited environments faced financial and logistical barriers, as highlighted by a provider from a non-profit organization:

*The biggest barrier is funding…. Traditionally the [in-person] fall prevention classes that we would offer in the community are all run by volunteers. (Exercise physiologist).*

Despite these challenges, providers leveraged teamwork and innovative strategies to maintain engagement with existing participants. However, reaching new clients remained difficult, as noted by a group fitness instructor:

*I have limited outlets. So, if people were not already involved in the program as a client, then all I can do beyond that is mentioned it to older friends of mine…. I can see someone thinking “I would do something like this if I knew it existed” so I’m just hoping that people who want to participate are finding out about it. (Group fitness instructor).*

#### 3.3.2 Theme 2: Tailoring remote supports to needs and abilities promoted safety

Ensuring participant safety was a central focus in the design and adaptation of remote supports, in particular virtual exercise programming. Exercise providers implemented several precautionary measures such as structured warm-ups, space preparation guidelines, and real-time monitoring strategies to safeguard participants and minimize potential risks. One instructor outlined these safety considerations:

*We have some defined strategies to minimize risk and increase safety… always doing a warm-up and cool down, always instructing people to prepare their space at home, ensuring that they are obstacle free, letting them know if you do feel chest pain or light headedness or dizziness that they stop and let us know right away… things to help build awareness and monitoring. (Group fitness instructor).*

Tailoring exercises to match participants’ abilities was critical, but assessing new clients remotely posed challenges and was sometimes deemed impractical, particularly for older adults with complex health conditions. A kinesiologist expressed concerns about the limitations of virtual assessments:

*I do not feel comfortable at this point doing a remote assessment on post-stroke individuals. Many of them have significant movement dysfunction and aphasia as well, which makes the communication piece significantly harder. To do that remotely is a big challenge. (Kinesiologist).*

Safety concerns about supervising exercise remotely were less prominent with real-time virtual programs as exercise providers could assess the participant’s exercise space and offer immediate feedback. To address safety concerns, providers recognized that smaller class size or virtual breakout rooms with at least one exercise provider in each room helped to reduce the client-to-leader ratio. The use of large monitors also afforded a better view of clients’ exercise movements.

Some providers also adapted exercise routines to account for participants’ available space and equipment for enhanced safety. Some organizations supplied small exercise equipment (e.g., resistance bands) while others used bodyweight exercises or household items:

*We planned the exercises to be able to be done with minimal equipment…one of the first things we did was make a video that talked about common household items that can be used for resistance training. (Kinesiologist, Exercise physiologist).*

#### 3.3.3 Theme 3: real-time virtual programs fostered social support and engagement

Beyond physical benefits, real-time virtual programs served as a vital source of social connection and emotional support for participants. Real-time sessions were particularly effective in fostering engagement, with many exercise providers reporting that older adults experienced improved mood and motivation as a result:

*Classes have really lifted their spirits and helped with motivation…the live sessions have been the most successful in creating those social connections. It’s not the same as in-person, but I do think it’s helped people whose mental health has been impacted negatively. (Exercise physiologist).*

Consistent interactions with instructors and peers created a sense of community, reinforcing participation. These interactions also provided exercise providers with valuable insight into older adults’ experiences with remote support and at-home exercise, with regular feedback affirming that the sessions helped older adults feel reconnected and energized. However, social engagement was more limited for individuals using pre-recorded sessions, making follow-up efforts challenging:

*The struggle is the people who are only opting into the pre-recorded sessions. We do not have the same level of communication with them, so we cannot follow up. We’ll send emails out… if they do not correspond to our group emails, our ability to know how they are doing is certainly dampened compared to the involvement we have with those that have opted into the live stream sessions. (Group fitness instructor).*

To mitigate this, providers explored alternative communication methods, such as phone check-ins, though the perceived effectiveness of this approach was varied.

#### 3.3.4 Theme 4: accessible technology and ongoing support facilitated virtual delivery

While virtual exercise provided an accessible alternative to in-person programming, the transition to virtual exercise was not without obstacles. With many older adults facing technological barriers and requiring support to navigate online platforms, planning for virtual delivery required thoughtful decision-making, training, and privacy safeguards. User-friendliness and cost were also key factors when selecting delivery platforms. Organizations implemented step-by-step guidance, instructional videos, and real-time assistance to facilitate participation in virtual exercise programs:

*Each week we would take them through one step of the process with detailed instructions, and sometimes how-to videos that would also guide them. One week it was making their [email] accounts, the next week it was logging into [virtual platform name], and then the next week it was how to use the platform and sending out those invites. (Exercise physiologist).*

Collaboration with community resources, such as libraries, further helped address digital literacy challenges. However, reliable internet access remained a barrier, particularly in rural areas or among participants with financial constraints. In some cases, providers explored alternative solutions, such as telephone-based support. This approach enabled individuals to participate in exercise programs that would otherwise be inaccessible due to technological and/or internet limitations, particularly benefiting older adults in rural areas with limited connectivity and those with specific health challenges, as noted by one provider:

*There’s one participant who kept repeatedly telling me “I really hope that once this all goes back to normal that you guys keep making phone calls. There’s an exercise program…in the bottom of my building and I cannot go.” Because she was visually impaired, she has no way to get there even though it’s in her building. (Group fitness instructor).*

Privacy concerns were less frequently reported but were addressed through platform security measures, private session links, and optional virtual backgrounds.

#### 3.3.5 Theme 5: a hybrid approach balances convenience and social benefits

Looking beyond the pandemic, providers anticipated a future where hybrid models—combining virtual and in-person exercise — would become the norm. Virtual sessions offered unparalleled convenience, particularly for older adults facing mobility challenges, caregiving responsibilities, or adverse weather conditions:

*One of the nice things about it [remote programming] is that nobody has to leave their home. Some people have already said that even if it were not for the pandemic, in the wintertime they would really appreciate being able to take a class online. (Group fitness instructor).*

For some older adults, virtual exercise provided a sense of independence and flexibility. One instructor described how a participant caring for her husband with dementia used virtual classes as a moment of respite:

*She said that it feels like her little break of freedom because she can exercise and he’s just so busy watching [the exercise videos]. so she gets a little bit of a break to do her exercise which is something that she enjoyed. (Group fitness instructor).*

Exercise providers also found flexibility in delivering remote classes, benefiting both their participants and themselves. Some were able to conduct sessions from home or, if needed, from the office, with setups designed to accommodate either location. Despite these benefits, maintaining motivation in a virtual setting was a challenge. In-person programs facilitated social accountability, which was harder to replicate in remote formats:

*When they [clients] are coming in-person, as much as it is exercise for them, for a lot of them it’s the social factor too, so without that drive to get somewhere to be, it’s a lot more challenging for a lot of people to have the motivation to do things from home. (Kinesiologist).*

The hybrid approach was seen as an adaptable solution, offering older adults the flexibility to maintain their fitness routines while balancing personal or logistical challenges. This approach provides the flexibility to maintain an exercise routine regardless of one’s location or personal circumstances: *“[with hybrid options] clients have access to everything in person at the facility, but they also have the option to do classes virtually and still stay active.” (Kinesiologist).*

Ultimately, while virtual methods proved to be a valuable tool, the sustainability of a hybrid model depended on the capacity of exercise providers to manage both in-person and remote offerings effectively.

Overview table provides a summary of providers’ recommendations for successful remote exercise delivery (Table 4).

**Table 4:** Overview table: summary of exercise providers’ tips for virtual programming.

Theme/Domain	Recommended strategy
Pre-program startup	Assess participants’ potential barriers before program launch (e.g., technology access, digital literacy, health concerns). Offer technology training sessions with step-by-step guidance, including video tutorials and/or written instructions. Conduct individual or small-group check-in calls to support onboarding and troubleshoot early challenges. Encourage participants to prepare their exercise space for safety (e.g., removing obstacles, securing furniture).
Program design and delivery	Tailor class intensity and content to participants’ fitness levels, health conditions, and available home space or equipment ensuring accessibility through real-time feedback. Incorporate structured on-screen warm-ups and cool-downs using clear visual and verbal cues to promote safety and reduce injury risk in home environment. Adapt exercises for minimal equipment use, offering modifications for those with limited space or physical constraints. Maintain small class sizes or use breakout rooms in virtual settings to ensure individualized support.
Fostering engagement and social connection	Integrate social opportunities into sessions (e.g., icebreakers, post-class discussions, peer interactions). Prioritize real-time virtual sessions when possible to enhance motivation, accountability, and a sense of community. Provide follow-up communication (e.g., emails, phone check-ins) to maintain engagement, particularly for those using pre-recorded or phone-based programming.
Technology accessibility and adaptation	Select user-friendly, reliable, and cost-effective platforms, ensuring compatibility across devices (computer, tablet, smartphone, or TV). Offer alternative participation methods, such as phone-based exercise guidance for those with limited internet access. Address privacy concerns by guiding participants on security settings (e.g., using virtual backgrounds, restricting access to live sessions).
Ensuring safety in remote exercise	Provide clear verbal and visual instructions to support safe execution of exercises. Encourage participants to listen to their bodies and recognize warning signs (e.g., dizziness, chest pain) during exercise. Have a plan in place for handling emergencies in virtual sessions, such as a check-in system for at-risk participants.
Supporting long-term participation	At-home programs offer convenience and accessibility, while in-person classes encourage social connection and provide better oversight. Hybrid models can balance accessibility and social engagement. Only offer at-home options for those who can safely participate in unsupervised exercise, ensuring they have proper guidance. Maintain ongoing feedback loops with participants to refine program offerings based on their evolving needs and preferences.

## 4 Discussion

In this study, exercise providers viewed remote exercise supports as valuable tools for maintaining physical and mental well-being among older adults. Many envisioned a future that combined virtual and in-person options into a hybrid approach. Real-time virtual classes were considered the most effective remote support, as they fostered safety and a sense of community. Providing technical assistance to older adults was crucial, as the success of virtual programming often relied on clients’ technical knowledge and confidence.

### 4.1 Social interaction with virtual exercise for enjoyment and adherence

Our findings highlighted the value of embedding social interaction into real-time virtual exercise programs to boost enjoyment and adherence among older adults. Existing literature confirms that social contact with peers and exercise providers is a key motivator for physical activity among older adults (12, 50–52), with group settings fostering a sense of belonging and accountability (53, 54). Social engagement during physical activity may be a greater motivation than the activity itself (12, 55). Although interactive virtual programs can offer a social atmosphere, they may not fully replicate in-person connections and motivations. Further, when not integrated, the absence of social interaction may hinder exercise enjoyment and motivation (56, 57). As the pandemic progressed, pre-recorded exercise programs gained traction among providers, offering a scalable alternative to real-time virtual sessions (58). This shift, while broadening access, amplified concerns that pre-recorded videos may not offer the same motivational boost due to the lack of social engagement in this format. In this study, the perception that non-interactive options were less effective may stem from the absence of key factors identified by participants as essential for sustained exercise adoption, including social connection, communication, and a sense of community.

### 4.2 Exercise supervision and clinical populations

Considerations for participant safety emerged as a focus among exercise providers. Similar to in-person programs (59, 60), supervision during virtual exercise can offer a perceived feeling of safety among participants, with the potential to enhance motivation and program adherence. Virtual supervision also allows program leaders to adapt exercise programming to participant needs. Exercise supervision is particularly crucial for certain clinical populations; therefore, at-home exercise may not suit everyone. While at-home and hybrid programs have proven effective for cardiac rehabilitation (61), clinical populations requiring hands-on supervision or specialized equipment may find some remote approaches impractical or unsafe (60, 62). Previous remote exercise studies that offered only pre-recorded sessions to people with stroke (63) or Parkinson’s disease (64) reported low attendance rates, partly due to the lack of supervision and personalized feedback.

### 4.3 Technology as a facilitator and the promise of hybrid exercise programming

In this study, technology played a key role in facilitating remote exercise programs, enabling older adults to engage in physical activity from home while maintaining real-time interactions with trainers and peers for socialization and personalized guidance. Previous studies have highlighted the importance of convenience (65–67) and socialization (67, 68) in driving the adoption of technology-based exercise programs. Consistent with this, the exercise providers in this study favored real-time, virtual exercise programming as the most effective format.

However, limited technical skills and digital literacy emerged as significant barriers to engagement among older adults, aligning with provider concerns about digital literacy and technology adoption (67). Similarly, previous research has found that physiotherapists often avoided incorporating advanced technologies into at-home rehabilitation due to perceived limitations in participants’ technical abilities (69). Indeed, even at a time when technology was highly utilized during the COVID-19 pandemic, a study by Mehrabi et al. (67) found that while many older adults increased their technology usage to engage in remote exercise programming, those with limited prior experience often faced frustration and relied on family or peers for technical support. Despite these barriers, studies suggest that when older adults have prior technological experience or access to support, they demonstrate increased self-efficacy with technology, highlighting the potential for targeted interventions to reduce digital literacy barriers (67). Literature often highlights ease of use as a critical factor for home-based programs, especially for individuals with limited technology experience (65–68). A recent scoping review further supports this, revealing that older adults value and are willing to use technology for home-based exercise, provided they receive adequate support (70). Furthermore, intrinsic motivation – particularly the desire to maintain physical and mental health – can further support and drive technology adoption among older adults, even for individuals with minimal technology access or digital skills (67). As such, successful integration of technology into exercise programming requires user-friendly platforms, proactive technical assistance, and preparatory sessions to enhance uptake, build confidence, and sustain participation (71).

Reliable internet access was also identified as an essential component for virtual program implementation. In Canada, over 1 million rural households lack broadband internet (72), disproportionately affecting older adults, 23.2% of whom live in rural areas (73). This digital divide limits access to at-home exercise programming and makes telephone-based alternatives more viable.

Exploring a hybrid approach that combines in-person and remote delivery received highly favorable feedback from participants, with enhanced program accessibility frequently cited as a key benefit. Hybrid models offer the potential to overcome barriers that traditionally hinder older adults’ participation in physical activity programs, such as transportation difficulties, inclement weather and time constraints, by offering flexible delivery options that integrate the convenience of at home sessions with the structured support of in-person training (12, 67, 74–76). A recent systematic review and meta-analysis (75) found that remotely supervised exercise interventions delivered via videoconferencing are both feasible and effective for improving physical function, muscle strength, emotional well-being, and quality of life in older adults. Notably, the outcomes were comparable to those of in-person programs, particularly when real-time interaction with exercise professionals was maintained (75).

However, challenges remain for exercise adoption and sustainability in a hybrid program. Previous research has reported dropout rates as high as 30% in hybrid exercise programs, primarily due to lack of supervision and social interaction during the at-home sessions (77). In contrast, Kraal et al. (78) compared home-based and center-based rehabilitative exercise programs and found similar levels of program adherence in both groups, possibly due to the high degree of trainer contact and individualization in both settings (78). Together, these findings suggest that the feasibility and effectiveness of hybrid exercise programs hinge on balancing accessibility with strong professional support structures to promote adherence and optimize health outcomes.

Our study employed a mixed-method approach, combining quantitative data with qualitative insights to understand how exercise providers supported at-home exercise during the pandemic. Quantitative data offered measurable feedback that explored the utilization and efficacy of physical activity supports, while the qualitative findings provided rich information and context into how exercise providers engaged with virtual exercise and other remote physical activity programs. Taken together, this information provides valuable guidance for future home-based exercise programming. However, several study limitations should be noted. First, our sample was likely skewed toward exercise providers who were more technologically savvy. Social media recruitment may have further biased participation, as active users tend to be more comfortable with technology than those who use it less frequently. Additionally, the overall sample of both the survey and interviews were relatively small, predominantly female, and fluent in English. As such, our findings may not fully capture the perspectives of the broader, more diverse population of exercise providers across Canada and internationally. While a number of survey respondents expressed interest in a follow-up interview, only a portion were ultimately available. The limited participation likely reflects the heightened demands and capacity challenges faced by exercise providers during the COVID-19 pandemic. As a result, the qualitative findings reflect the perspectives of those who were both willing and able to engage within the study timeframe and may not fully represent the wider range of views and experiences within the field. Finally, all participants in our interviews reported very good to excellent physical and mental health, considerably higher than our survey sample. This suggests that interview data may be more biased than survey data.

## 5 Conclusion and implications

During the early months of the pandemic, exercise providers rapidly adopted remote methods to sustain older adults’ physical activity, navigating a landscape of trial and error. A clear preference emerged for real-time virtual programming, valued for its ability to provide oversight and foster social connections—key elements for engagement and safety. While technological barriers, such as limited digital literacy and access, posed significant challenges, the widespread adoption of digital tools throughout and beyond the pandemic has likely mitigated some of these hurdles. Given that aging populations and rural communities continue to face access disparities exacerbated by the digital divide, these findings remain highly relevant. Future research should prioritize the implementation and evaluation of hybrid physical activity programs, assessing their acceptability, adoption, and optimal design. Such models offer a promising way to merge the convenience of remote options with the community benefits of in-person settings, creating flexible and equitable pathways for older adults to sustain lifelong physical activity and well-being in an increasingly digital world.

## Data Availability

The quantitative dataset is available from the corresponding author upon reasonable request. However, the qualitative data, due to the highly personal nature of the responses and the risk of disclosure, are not publicly available. They can be obtained from the corresponding author upon reasonable request.
